# The relevance of uniform reporting in oral leukoplakia: Definition, certainty factor and staging based on experience with 275 patients

**DOI:** 10.4317/medoral.18756

**Published:** 2012-10-20

**Authors:** Elisabeth R E A. Brouns, Jacques A. Baart, Elisabeth Bloemena, Hakki Karagozoglu, Isaäc van der Waal

**Affiliations:** 1 VU University Medical Center (VUmc)/Academic Centre for Dentistry Amsterdam (ACTA), Department of Oral and Maxillofacial Surgery and Oral Pathology, P.O. Box 7057, 1007 MB Amsterdam, The Netherlands; 2 ……..; 3 ….

## Abstract

The aim of the present study was to evaluate the definition of oral leukoplakia, proposed by the WHO in 2005 and taking into account a previously reported classification and staging system, including the use of a Certainty factor of four levels with which the diagnosis of leukoplakia can be established. In the period 1997-2012 a hospital-based population of 275 consecutive patients with a provisional diagnosis of oral leukoplakia has been examined. In only 176 patients of these 275 patients a firm diagnosis of leukoplakia has been established based on strict clinicopathological criteria. The 176 patients have subsequently been staged using a classification and staging system based on size and histopathologic features. For use in epidemiological studies it seems acceptable to accept a diagnosis of leukoplakia based on a single oral examination (Certainty level 1). For studies on management and malignant transformation rate the recommendation is made to include the requirement of histopathologic examination of an incisional or excisional biopsy, representing Certainty level 3 and 4, respectively. This recommendation results in the following definition of oral leukoplakia: “A predominantly white lesion or plaque of questionable behaviour having excluded, clinically and histopathologically, any other definable white disease or disorder”. Furthermore, we recommend the use of strict diagnostic criteria for predominantly white lesions for which a causative factor has been identified, e.g. smokers’ lesion, frictional lesion and dental restoration associated lesion.

** Key words:**Oral epithelial dysplasia, oral leukoplakia, potentially malignant oral disorders.

## Introduction

The estimated prevalence of oral leukoplakia worldwide is approximately 2%,(1) with an annual malignant transformation rate into oral squamous cell carcinoma (OSCC) of approximately 1%. (2) In 1978, oral leukoplakia was defined by the World Health Organization (WHO) as: ‘a white patch or plaque that cannot be characterized clinically or pathologically as any other disease’. (3) At an international conference in 1984, an addition was made to the 1978 WHO definition: ‘oral leukoplakia is not associated with any physical or chemical causative agent except the use of tobacco’.(4) Thereafter, in 1986, the definition of oral leukoplakia has been changed into: ‘a predominantly white lesion of the oral mucosa that cannot be characterized as any other definable disease’. (5) In 1997, the phrase: ‘any other definable disease’ was replaced by ‘any other definable lesion’. (6)

In 2002, it was recommended to make a distinction between a provisional clinical diagnosis of oral leukoplakia and a definitive one. (7) A provisional diagnosis was made when a lesion at the initial clinical examination could not be clearly diagnosed as either leukoplakia or a definable lesion. In case of a provisional clinical diagnosis, Certainty factor 1 was assigned. A definitive clinical diagnosis of leukoplakia was made after unsuccessful elimination of suspected aetiological factors or in the absence of such factors, assigning Certainty factor 2. Certainty factor 3 was assigned when histopathological examination of an incisional biopsy did not show the presence of a definable lesion. In case of an excisional biopsy or surgical excision, performed after an incisional biopsy, Certainty factor 4 was assigned based on histopathological examination of the surgical specimen.

In 2005, the definition was changed by the WHO into: ‘a white plaque of questionable risk having excluded (other) known diseases or disorders that carry no increased risk for cancer’. (8) A list of definable white diseases and disorders, that may occur in the mouth, is presented in  Table 1. In case of a predominantly red appearance, the term erythroplakia is applied. This entity will not be discussed here any further.

**Table 1:**
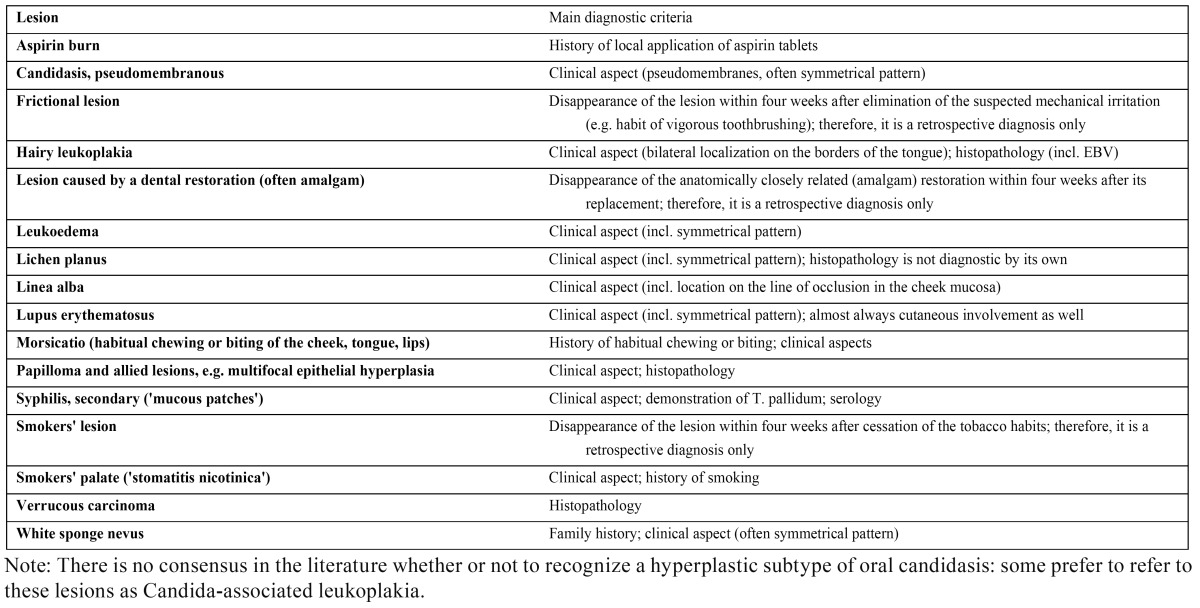
Definable white diseases and disorders that may occur in the mouth.

The aim of the present study was to evaluate the definition of oral leukoplakia, proposed by the WHO in 2005 and taking into account the previously mentioned classification and staging system ( Table 2), including the use of a Certainty factor ( Table 3). The management results in these patients, including the issue of malignant transformation, will be reported separately.

**Table 2:**
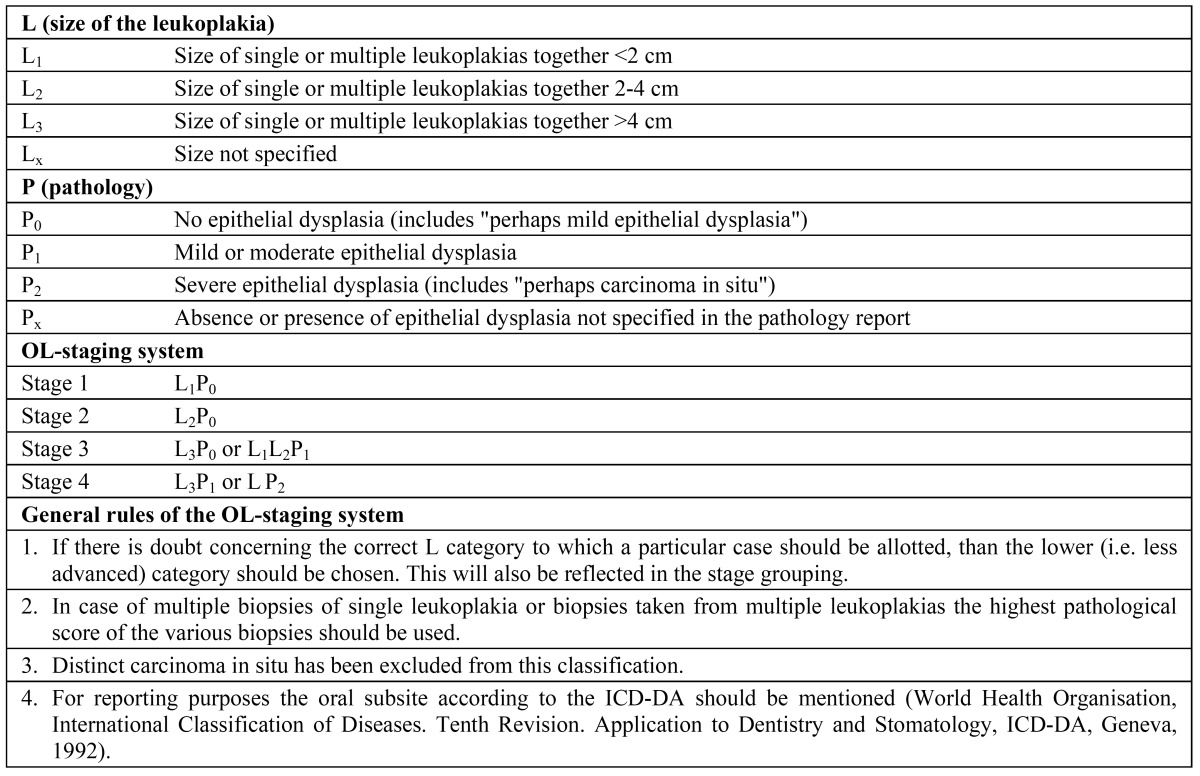
Classification and staging system for oral leukoplakias (OL-system)2.

**Table 3:**
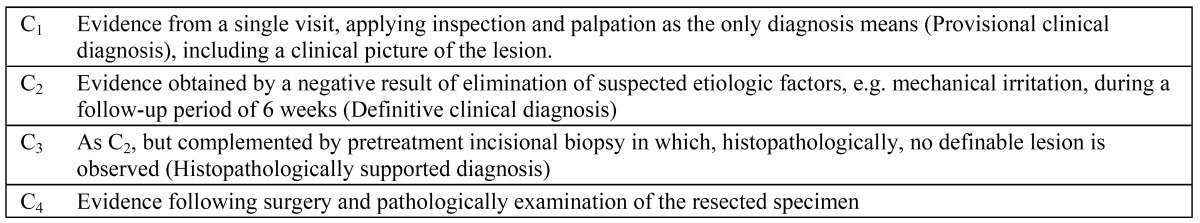
Certainty (C)-factor of a diagnosis of oral leukoplakia2.

## Material and Methods

-Patients

For the purpose of this retrospective study, 295 consecutive patients were included with a provisional clinical diagnosis of oral leukoplakia and being documented with at least one clinical picture at admission. In this study, oral leukoplakia is defined according to the WHO 2005 definition. All patients were referred to the Department of Oral and Maxillofacial Surgery and Oral Pathology at VUmc/ ACTA, Amsterdam, The Netherlands between 1997 and 2012. Exclusion criteria were patients with a previous or concomitant OSCC (n = 20). The remaining group of 275 patients consisted of 112 men and 163 women, with a mean age of 57 years (range 17-98 years).

In 171 patients a possible causative factor was identified, such as a dental restoration (“contact lesion”), mechanical irritation (“frictional lesion”) or tobacco use (“smokers’ lesion”), as being defined in  Table 1. A maximum of four weeks was observed for evaluation of the result of cessation of the suspected causative factor. The use of tobacco and alcohol was registered in a simpli-fied manner as being user, nonuser or unknown.

The location of the leukoplakia was specified according to eight subsites: 1) tongue, 2) floor of mouth (FOM), 3) lower lip, 4) hard palate, 5) buccal mucosa, 6) upper alveolus and gingiva, 7) lower alveolus and gingiva and 8) multiples sites ( Table 4).

**Table 4:**
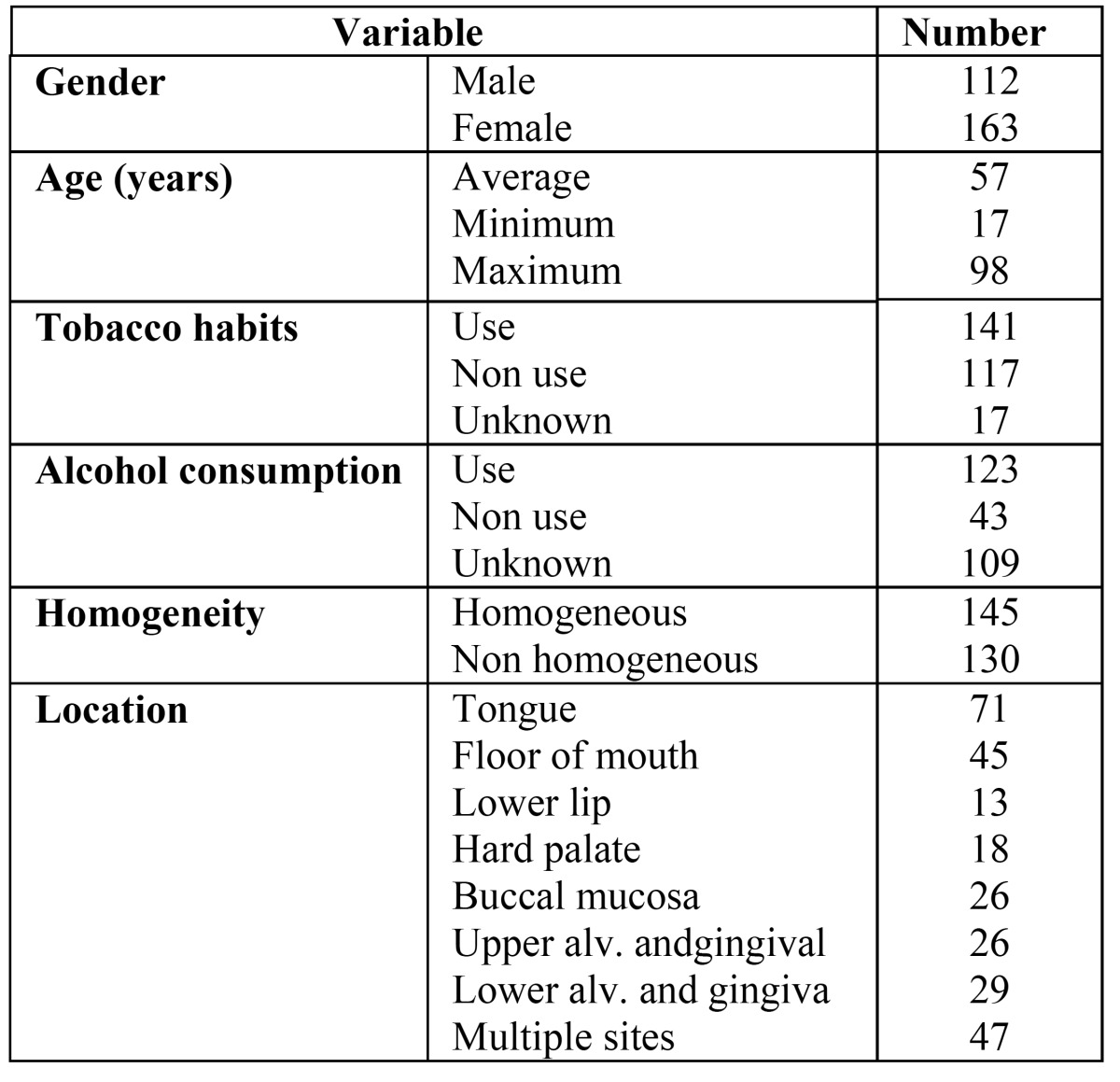
Demographic, aetiologic and clinical data of 275 patients with a provisional clinical diagnosis of oral leukoplakia (Certainty factor 1).

Clinically, a distinction was made between homogeneous (flat, thin, uniform white in colour) and nonhomogeneous (white or white-and-red, - erythroleukoplakia - either speckled, granular, nodular or verrucous) ( Table 4). (2) Proliferative verrucous leukoplakia (PVL) is a subtype of verrucous leukoplakia, being characterized by multifocal presentation, resistance to treatment and a high rate of malignant transformation.(9) This subtype will not be discussed here any further in detail.

**Figure 1:**
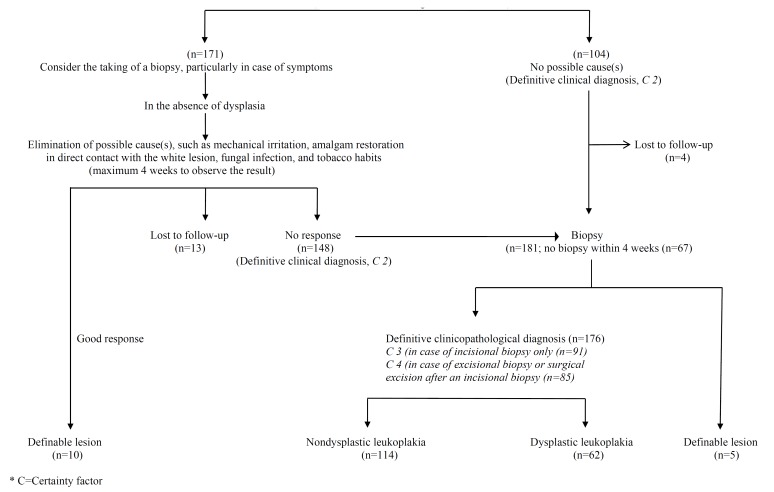
DIAGNOSIS OF ORAL LEUKOPLAKIA* (Provisional clinical diagnosis, C 1; n=275).

In all cases of a definitive clinical diagnosis of leukoplakia, the taking of a biopsy was recommended not later than four weeks (Fig. 1). The absence or presence of epithelial dysplasia has been recorded in three categories, being 1) no epithelial dysplasia or perhaps mild epithelial dysplasia (P0), 2) mild or moderate epithelial dysplasia (P1), and 3) severe epithelial dysplasia, possibly carcinoma in situ (P2). The size, presence and grade of epithelial dysplasia, if present, were grouped by means of the OL-classification and staging system ( Table 2).

The design of this study adheres to the code for proper secondary use of human tissue of the Dutch Federation of Biomedical Scientific Societies (http://www.federa.org). (10)

## Results

In this study, 275 patients with a provisional clinical diagnosis of oral leukoplakia were included (Certainty factor 1). There were 171 white lesions with possible causes, such as mechanical irritation, direct contact with a dental restoration or tobacco habits. In 10 patients a distinct regression or even complete disappearance of the lesion was observed. Thirteen patients did not show up for the assessment of the elimination of these possible causes. In the remaining 148 patients no response was observed, resulting in a definitive clinical diagnosis (Certainty factor 2). In 104 patients there were no possible causes, also resulting in a definitive clinical diagnosis (Certainty factor 2); four of these patients did not show up for the taking of a biopsy. As a result 248 patients were diagnosed with a Certainty factor 2.

Of the 248 patients with a definitive clinical diagnosis of oral leukoplakia, a biopsy was taken in only 181 patients. In five patients the histopathological examination resulted in a “definable lesion” ( Table 1), including two cases of (verrucous) squamous cell carcinoma. In the remaining 176 patients, 109 incisional biopsies (Certainty factor 3) and 67 excisional biopsies (Certainty factor 4) were performed.

Of the 109 patients in whom an incisional biopsy was taken, 34 showed various degrees of epithelial dysplasia, while in 75 patients no dysplasia was observed. In these patients treatment consisted of additional surgical excision (n = 18), CO2 laser vaporisation (n = 33) and observation (n = 58). In case of additional surgical excision a specimen was available for additional histopathological examination. In five of the 18 patients the final histopathological examination resulted in a different P-classification; four patients were upgraded from stage 1 to stage 3, while one patient was downgraded from stage 3 to stage 1.

In 67 patients in whom an excisional biopsy was taken, various degrees of epithelial dysplasia have been observed in 28 patients. In the remaining 39 patients no dysplasia was observed.

In the 67 patients in whom no possible cause could be identified, no biopsy was taken within a period of four weeks after the first admission. In some of these patients a biopsy was taken at a later stage, while others were lost to follow-up.

The results of the diagnostic process are shown in (Fig. 1), while the results of the OL-staging are shown in ( Table 5).

**Table 5:**
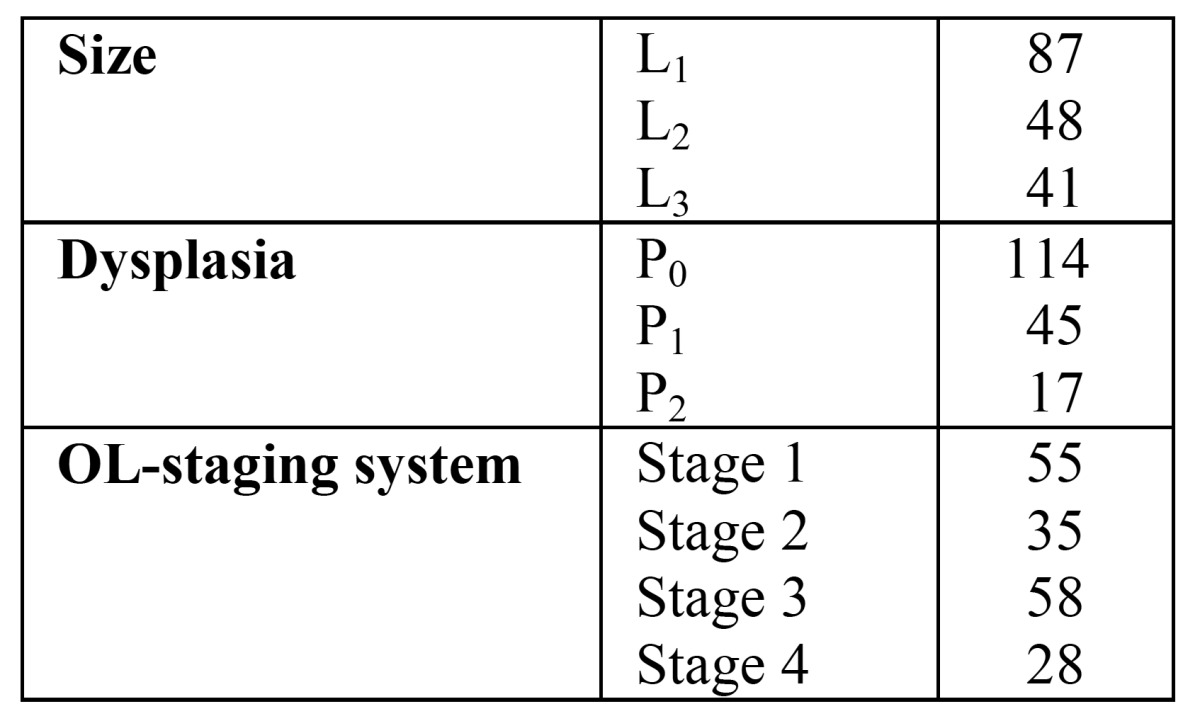
Size, histopathology and stage in 176 patients with a definitive clinicopathologic diagnosis of oral leukoplakia (Certainty factor 3 or 4).

## Discussion

In the present study, 275 patients with a provisional clinical diagnosis of oral leukoplakia were included (Certainty factor 1). Based on strict definitions only 176 of the initially 275 patients have been diagnosed with a definitive clinicopathological diagnosis of oral leukoplakia (Certainty factor 3 or 4). Therefore, the use of strict criteria for the diagnosis of leukoplakia is recommended in reporting treatment results and also in reporting the results of epidemiological studies.

The taking of a biopsy should be considered before the attempt of elimination of a possible cause, particularly in case of symptoms. In the past, we have encountered, indeed, an occasional patient with a clinical diagnosis of leukoplakia in whom a squamous cell carcinoma was already present in the biopsy taken at the first admission.

It is known that leukoplakia in patients with tobacco habits might be reversible if patients give up their smoking habits. (11,12) Therefore, these patients should be advised to give up their habits. If successful and if the white lesion regresses within a somewhat arbitrarily chosen period of no more than four weeks the provisional clinical diagnosis of such lesion should, in retrospect, be changed into “smokers’ lesion”. It is well appreciated, that it actually may take more than four weeks for such a smokers’ lesion to completely disappear. Others have shown resolvement of these lesions after cessation of the tobacco habits for six weeks. (13) A longer period of observation may be allowed provided a prior biopsy has not shown the presence of any degree of epithelial dysplasia. When the patient is not able or willing to give up the tobacco habits and in persistence of the lesion, the relevance of applying the term “tobacco-associated leukoplakia” seems questionable.

Another possible reversible white lesion is the frictional lesion caused by mechanical irritation. This lesion is sometimes referred to as “frictional keratosis”. (14-16) The term “lesion” is preferred because “keratosis” is actually a histopathological term. Similarly as in smokers’ lesion we recommend to restrict a final diagnosis of frictional lesion to cases where the lesion has disappeared after elimination of the mechanical cause, within a somewhat arbitrarily chosen period of no more than four weeks. As in a smokers’ lesion, the definitive diagnosis of frictional lesion can only be made in retrospect. The same applies to “contact lesion” caused by amalgam.

Furthermore, there is room for discussion about the diagnosis candidiasis versus Candida-associated leukoplakia, particularly if located at the commissures of the lips and the dorsal surface of the tongue. If such lesions regress or disappear after antifungal treatment within the previously mentioned period of four weeks there is no justification to call such lesions leukoplakias any longer. However, in case of persistence, the diagnosis of (Candida-associated) leukoplakia remains valid.

Although alcohol might play a role in the aetiology of oral leukoplakia, no studies are available in which the results of cessation of alcohol habits as a single factor has been examined. Perhaps there is such an entity of a leukoplakialike lesion caused by alcohol (“alcohol lesion”) that disappears after quitting the habit.

In five out of 181 patients with a definitive clinical diagnosis of leukoplakia the final diagnosis has been changed into papilloma, cheilitis actinica, verrucous carcinoma, squamous cell carcinoma, and lichen planus. As reported elsewhere, (17) we hesitate to accept a histopathological diagnosis of lichen planus as a final diagnosis. Rather, we prefer the description “compatible with lichen planus”. The issue of “lichenoid dysplasia” has been thoroughly discussed in the literature. (18) We discourage the use of such term since it, erroneously, may suggest dysplastic changes in oral lichen planus.

It is well recognized that the assessment of the presence and degree of epithelial dysplasia carries some subjectivity, reflected in a distinct intra- and interobserver variation. (19,20) In the present study, the absence or presence of epithelial dysplasia has been recorded into three categories, being 1) no or perhaps mild epithelial dysplasia (P0), 2) mild or moderate epithelial dysplasia (P1), and 3) severe epithelial dysplasia, possibly carcinoma in situ (P2) ( Table 2). We prefer a three category system above a four category system to reduce the intra- and interobserver variation between oral pathologists. Therefore, ‘perhaps mild epithelial dysplasia’ was included in the first grade. In the WHO monograph on Head and Neck Tumours severe epithelial dysplasia is recognized as a separate entity form carcinoma in situ. (21) In the present study we have followed these recommendations. In case of carcinoma in situ the diagnosis of leukoplakia is abandonned ( Table 2). Kujan et al. (22) applies only two histopathological grades, being 1) “low risk” (less than four architectural changes or less than four cytological changes), and 2) “high risk” (at least four architectural changes and five cytological changes). In this system, the authors used the architectural and cytological changes that have been described in the previously mentioned WHO monograph ( Table 6). (21) At present, there is insufficient justification to include one or more biological markers that may have predictive value in malignant transformation, such as DNA ploidy, in a staging system.

**Table 6:**
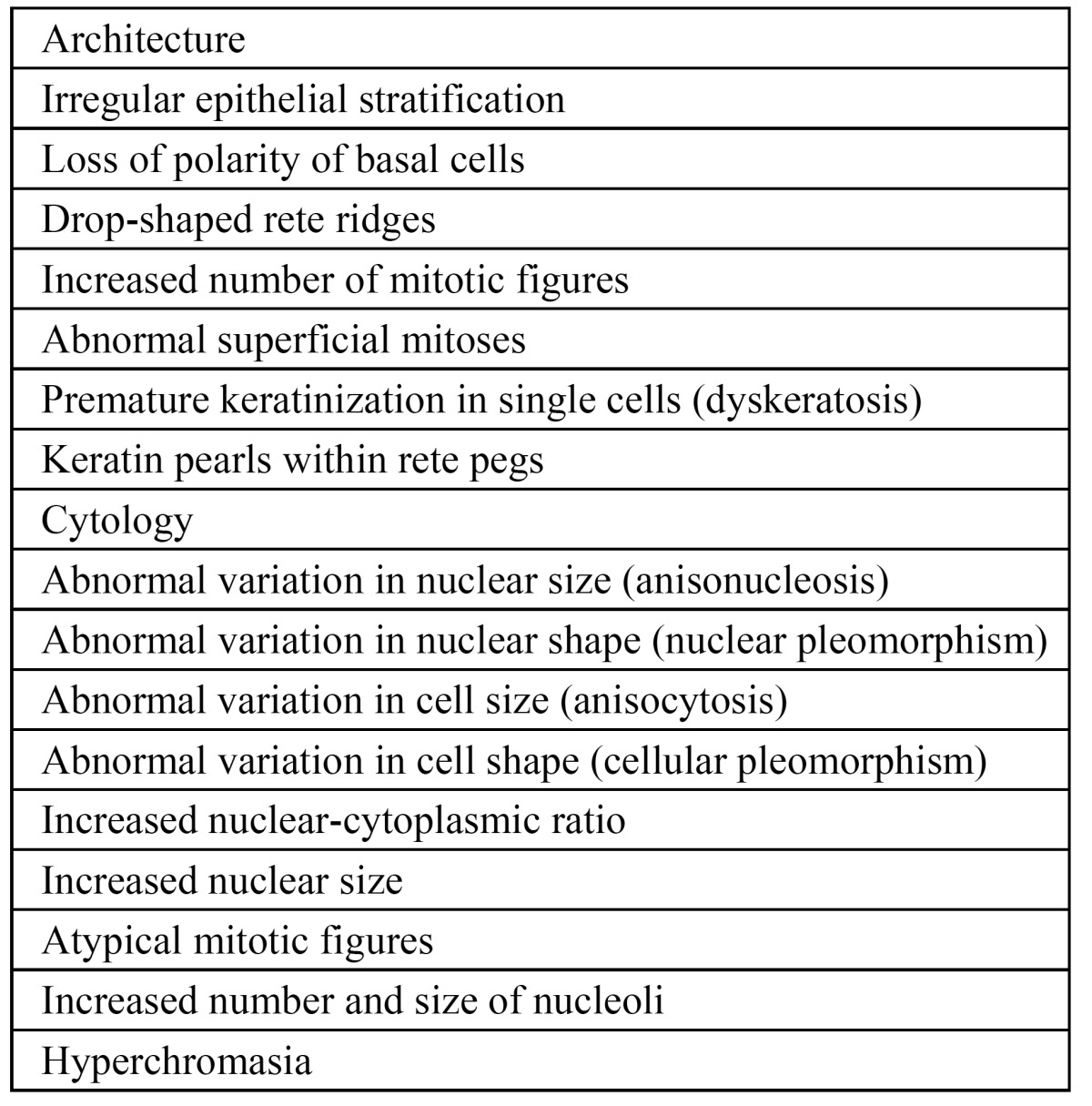
Criteria used for diagnosing dysplasia.21

In the 1978 WHO definition of oral leukoplakia it has been explicitly stated that the term leukoplakia is unrelated to the absence or presence of epithelial dysplasia. Therefore, we discourage the use of the term leukoplakia as a histopathological term. In case of a biopsy of a leukoplakia, the pathologist is advised to always report the absence or presence of epithelial dysplasia and its degree.

There are actually no strict criteria how to make a distinction clinically between verrucous leukoplakia and verrucous carcinoma. Histopathologically, several papers have been published about the histopathological difference between verrucous hyperplasia and verrucous carcinoma, still leaving room for discussion. (23) 

There is also room for discussion about the presently proposed classification of the size of a leukoplakia in three groups (< 2 cm, 2-4 cm, > 4 cm). In fact, the classification is more or less based on the T classification of oral squamous cell carcinoma. In a study by Holmstrup et al. a surface of 200 mm2 was shown to be of relevance with regard to the risk of malignant transformation. (24) Therefore, one might consider the use of a size classification in two groups, being < 200 mm2 or > 200 mm2.

In the sixties of the past century a minimum size of 5 mm was required before being allowed to use the term leukoplakia. (25) Although there may be some practical value in re-introducing a minimum size in the definition of leukoplakia, we hesitate to do so. Another part of previous definitions of leukoplakia has been the requirement of a nonremovable nature of the white lesion, apparently meant to separate pseudomembranous candidiasis from leukoplakia. The adjective “nonremovable” seems, indeed, to have some practical value, but we do not recommend its re-use at present.

One may consider to include the oral subsite in the staging system. For instance, in some studies leukoplakias of the floor of the mouth and the tongue have been reported to more often transform into malignancy than leukoplakias at other subsites. However, in other parts of the world, e.g. India, leukoplakias at the buccal mucosa are more at risk. Therefore, we do not recommend to include the subsite in the staging system.

The clinical distinction between homogeneous and nonhomogeneous leukoplakia has shown to be a valuable predictor of possible malignant transformation. (24) In daily practice it is difficult to always correctly make the distinction between these two types in a reproducible manner. The same applies to the various subtypes of nonhomogeneous leukoplakias, such as speckled, nodular, granular and also the already mentioned (proliferative) verrucous types. Nevertheless, we suggest to maintain these descriptions.

The adjectives “premalignant”, “precancerous” and “potentially malignant” designate the increased risk of malignant transformation. At present, there seem no strong reasons to change the WHO preference for the use of “potentially malignant”.

It is well appreciated that a number of aspects of the presently discussed definition and staging system may not be equally valid in all parts of the world. We have taken notice of a recently proposed classification from India for potentially malignant disorders of the oral cavity. (26) Obviously, this new classification is not limited to the presently discussed entity of leukoplakia, but also includes entities such as lichen planus, oral submucous fibrosis, nutritional deficiencies and some inherited cancer syndromes.

It is beyond the aim of the present study to further discuss the possible value of diagnostic adjuncts, e.g. toluidine blue. This also applies to discussion on the various treatment modalities and the malignant transformation rate. Suggestions for uniform reporting of treatment results have been discussed elsewhere. (2)

## Conclusions

Based on the results of the present study the use of strict diagnostic criteria for oral leukoplakia and allied white lesions is recommended. Furthermore, the presented classification and staging system seems valuable for comparing management results of patients with oral leukoplakia and also for use in epidemiological studies.

The recommendation is made to modify the present WHO definition of oral leukoplakia by adding the requirement of histopathologic examination in order to obtain a definitive clinicopathological diagnosis. As a result, we suggest: “A predominantly white lesion or plaque of questionable behaviour having excluded, clinically and histopathologically, any other definable white disease or disorder”. Furthermore, we recommend the use of strict diagnostic criteria for predominantly white lesions or diseases for which a causative factor has been identified, e.g. smokers’ lesion, frictional lesion and dental restoration associated lesion.
